# HiCube: interactive visualization of multiscale and multimodal Hi-C and 3D genome data

**DOI:** 10.1093/bioinformatics/btad154

**Published:** 2023-03-24

**Authors:** Tiantian Ye, Yangyang Hu, Sydney Pun, Wenxiu Ma

**Affiliations:** Department of Statistics, University of California Riverside, Riverside, CA 92521, United States; Department of Computer Science and Engineering, University of California Riverside, Riverside, CA 92521, United States; Department of Computer Science and Engineering, University of California Riverside, Riverside, CA 92521, United States; Department of Statistics, University of California Riverside, Riverside, CA 92521, United States

## Abstract

**Summary:**

HiCube is a lightweight web application for interactive visualization and exploration of diverse types of genomics data at multiscale resolutions. Especially, HiCube displays synchronized views of Hi-C contact maps and 3D genome structures with user-friendly annotation and configuration tools, thereby facilitating the study of 3D genome organization and function.

**Availability and implementation:**

HiCube is implemented in Javascript and can be installed via NPM. The source code is freely available at GitHub (https://github.com/wmalab/HiCube).

## 1 Introduction

Recent advances in high-throughput sequencing technologies have led to a vast accumulation of diverse types of genomics data. Among them, the Hi-C technique (Lieberman-Aiden et al. 2009) and its variants have provided unprecedented opportunities for studying the 3D genome organization and function. Integrative analyses of Hi-C and other genomics data have yielded better understanding of both the structural and functional roles of the chromatin (Zhang et al. 2022). Therefore, there exists a high demand to fast and interactively visualize and explore multimodal Hi-C and 3D genome data.

Recently, several visualization tools for Hi-C and 3D genome data have been developed (Supplementary Table S1). However, most of the existing tools focus on the visualization of 2D Hi-C contact maps together with 1D genomics, transcriptomics, and epigenomics tracks, while few pay attention to 3D genome structures. Currently, WashU Epigenome Browser (Li et al. 2022), Nucleome Browser (Zhu et al. 2022), TADkit (Serra et al. 2017), and HiC3D-Viewer (Djekidel et al. 2017) are the only tools that support simultaneous visualization of 2D Hi-C data and 3D genome structure data. However, WashU Epigenome Browser and TADkit cannot display inter-chromosomal interactions in Hi-C, and TADkit is no longer maintained. HiC3D-Viewer does not support zoom or pan operations to navigate the 2D Hi-C heatmap. In addition, both WashU Epigenome Browser and Nucleome Browser are designed for public datasets and web services, and therefore provide less local usage support and fewer types of user custom annotations.

Therefore, we developed HiCube, a lightweight web application that allows fast and interactive visualization and exploration of diverse types of genomics data (including 1D gene annotations, RNA-seq, ChIP-seq, ATAC-seq etc., 2D Hi-C contact maps, and 3D genome structures) at multiscale resolutions. We designed a user-friendly graphical user interface (GUI) for users to navigate (e.g. zoom or pan) on either genome-wide or chromosome-wide Hi-C maps, create annotations [such as topologically associating domains (TADs) and chromatin loops], configure display options, and export publication-ready images. HiCube can be installed locally to use private data, and it can load tracks from both remote servers and local storage. A detailed comparison between HiCube and other Hi-C and 3D genome visualization tools is available in Supplementary Table S1. In particular, two unique features of HiCube are worth noting: zoom view for multiscale visualization, and paired view instances for comparative visualization.

HiCube is implemented in Javascript and can be installed via NPM. The source code is publicly available at https://github.com/wmalab/HiCube.

## 2 Components and implementation

**Figure 1.**
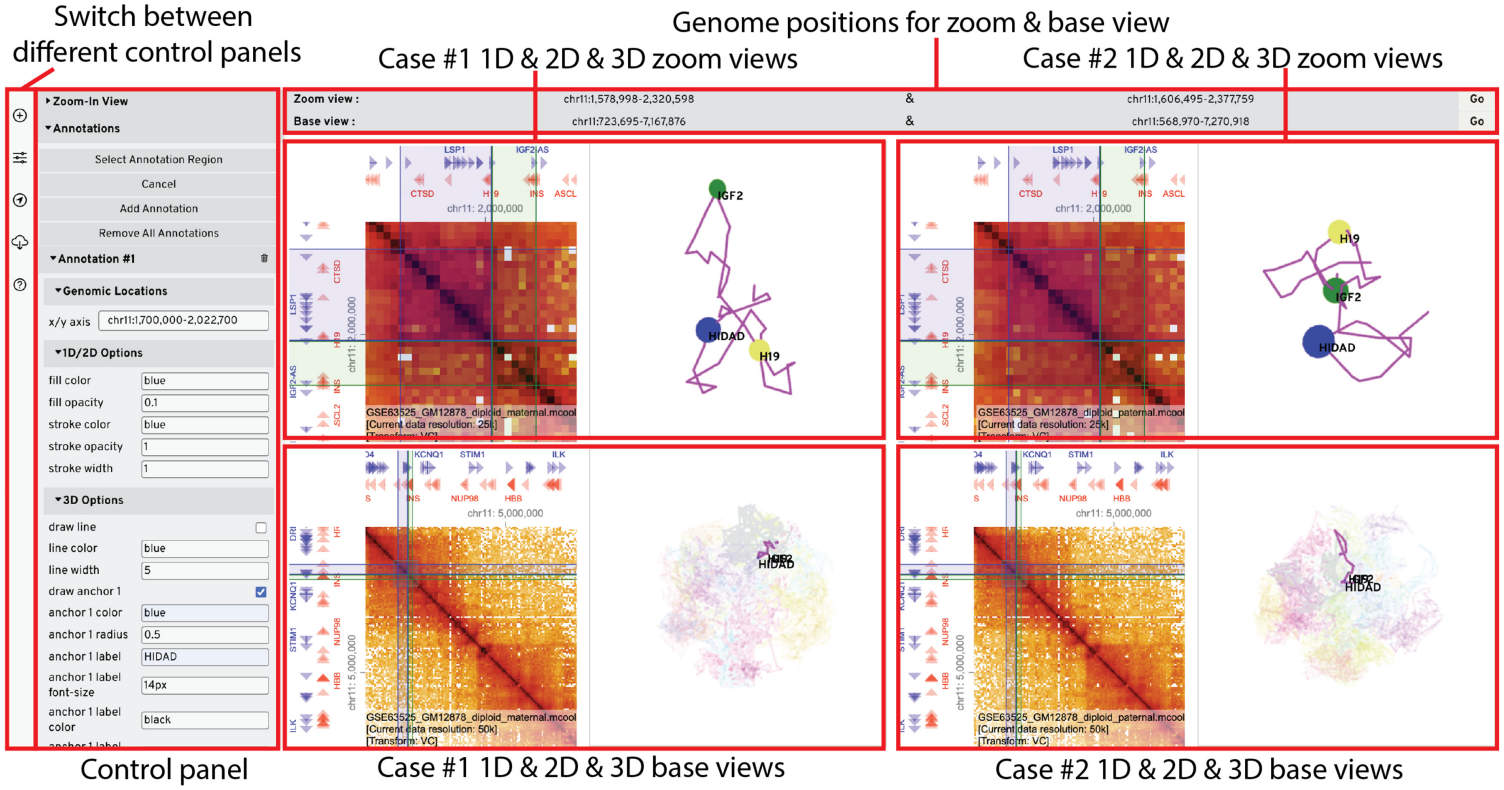
HiCube user interface. As an example, maternal (left, case #1) and paternal (right, case #2) difference at the local H19-IGF2 region (top, zoom view) and at global scale (bottom, base view) are displayed. The genome position bars on top display/change the current viewing regions for each view instances. The leftmost side is a set of buttons to switch between different control panels: add new case and tracks, edit track configurations, activate tools to select/create zoom view, or add/edit annotations, export configurations and figures, user guide

HiCube consists of three key modules: (i) visualization of 1D and 2D data, (ii) visualization of 3D data, and (iii) configuration panels (Fig. 1). The 1D and 2D visualization is implemented using HiGlass (Kerpedjiev et al. 2018), a fast visualization library for large-scale genomic data using tiling inspired by maps. The 3D visualization is implemented using Three.js, a Javascript library to display 3D graphics using WebGL. These two visualization components are unified under the React framework, which allows synchronization among 1D, 2D, and 3D view instances. The configure panels contain multiple user-friendly tools for adding data, changing display options, selecting zoom view regions, creating annotations, and exporting publication images.

HiCube takes three different types of input data: (i) 1D and 2D data in the tileset format, which can be created from cooler, bigWig, BED, BEDPE, BedGraph, etc. using HiGlass (Kerpedjiev et al. 2018). Users can either retrieve public datasets from the HiGlass API server, or load private datasets from local server using the HiGlass Docker container; (ii) 3D genome structure data in the g3d format (Li et al. 2022) from local storage; (iii) quantitative annotations, in the format of either genomic intervals (similar to BED) or paired-end genomic intervals (similar to BEDPE), with an optional score value to represent the signal intensity for 1D or 2D annotations, respectively. Users can create annotations from mouse selection, or enter/upload a list of intervals. Annotations are shown on all 1D, 2D, and 3D view instances. In particular, for quantitative annotation, users can visualize the signal intensity distribution on the 3D structure. A JSON format configuration file which defines all view instances can be downloaded, and loaded later to restore the view instances. User-selected annotations can also be exported for record keeping.

Two unique features of HiCube are the zoom view and paired view instances. Genome architecture is hierarchical; chromatins are organized at multiple scales from chromatin loops, to (sub-)TADs, A/B compartments, and chromosomes. To better illustrate the chromatin conformation at different scale levels, a zoom view can be created from mouse selection for 1D, 2D, and 3D view instances to display finer details such as loops and TADs at a higher resolution, while the default base view shows coarser features like A/B compartments at a lower resolution. Besides zoom view, another common demand is to compare genomics data side-by-side between different alleles, cells, or experimental conditions. Paired view instances can be utilized in such applications to facilitate comparative 3D genome analysis. After the view instance of the first condition is initialized, a second condition can be added, where every new track in the second condition is paired with its counterpart in the first condition. Later, any adjustments made on one view instance will be synchronized to the other, including changing base (or zoom) view regions, adding annotations, or configuring the display options such as height, width, color, etc.

Figure 1 illustrates an example of the HiCube user interface comparing the H19-IGF2 region on maternal (left) and paternal (right) chromosomes, where the genomic and 3D locations of the H19/IGF2 Distal Anchor Domain (HIDAD), H19, and IGF2 are labeled to show the differences in proximity between HIDAD and the others between the two parental chromosomes (Rao et al. 2014).

## Supplementary Material

btad154_Supplementary_Data

## References

[btad154-B1] Djekidel MN , Wang M, Zhang MQ et al HiC-3DViewer: a new tool to visualize Hi-C data in 3D space. Quant Biol 2017;5:183–90.

[btad154-B2] Kerpedjiev P , Abdennur N, Lekschas F et al HiGlass: web-based visual exploration and analysis of genome interaction maps. Genome Biol 2018;19:1–12. 30143029 10.1186/s13059-018-1486-1 PMC6109259

[btad154-B3] Li D , Purushotham D, Harrison JK et al WashU epigenome browser update 2022. Nucleic Acids Res 2022;50:W774–81. 10.1093/nar/gkac238 PMC9252771 35412637

[btad154-B4] Lieberman-Aiden E , van Berkum NL, Williams L et al Comprehensive mapping of long-range interactions reveals folding principles of the human genome. Science 2009;326:289–93. 19815776 10.1126/science.1181369 PMC2858594

[btad154-B5] Rao SSP , Huntley MH, Durand N et al A 3D map of the human genome at kilobase resolution reveals principles of chromatin looping. Cell 2014;159:1665–80. 25497547 10.1016/j.cell.2014.11.021 PMC5635824

[btad154-B6] Serra F , Baù D, Goodstadt M et al Automatic analysis and 3D-modelling of Hi-C data using TADbit reveals structural features of the fly chromatin colors. PLoS Comput Biol 2017;13:e1005665. 28723903 10.1371/journal.pcbi.1005665 PMC5540598

[btad154-B7] Zhang R , Zhou T, Ma J. Multiscale and integrative single-cell Hi-C analysis with Higashi. Nat Biotechnol 2022;40:254–61. 34635838 10.1038/s41587-021-01034-y PMC8843812

[btad154-B8] Zhu X , Zhang Y, Wang Y et al Nucleome browser: an integrative and multimodal data navigation platform for 4D nucleome. Nat Methods 2022;19:911–3. 35864167 10.1038/s41592-022-01559-3 PMC9357120

