# Supplementary material for: HiCube: interactive visualization of multiscale and multimodal Hi-C and 3D genome data
Source: Bioinformatics. 2023 Mar 24;39(4):btad154. doi: 10.1093/bioinformatics/btad154 (PMC10081873; doi:10.1093/bioinformatics/btad154)
Supplement: btad154_Supplementary_Data [file btad154_supplementary_data.pdf]

Supplementary Table S1: Feature comparison of HiCube with existing Hi-C and 3D genome visualization tools

Features	3D genome structure	2D Hi-C intra-chromosomal heatmap	2D Hi-C inter-chromosomal heatmap	1D tracks	Offline/local usage	Synchronization between samples	Zoom view	Annotate 1D regions	Annotate 2D regions
HiCube	Choose between different resolutions and categories, show in genome-wide, chromosome-wide or only current viewing region, show 1D or 2D annotations with texts, show 1D or 2D quantitative annotations, set the transparency of the unvisualized chromosomes, set the chromosome colors	Freely zoom & pan at multiple resolutions genome-wide, zoom & pan chromosome-wide, set width and height of the heatmap, set width/height ratio by entering genomic regions	Freely zoom & pan at multiple resolutions genome-wide, zoom & pan chromosome-wide	Gene annotations, gene expression data (RNA-seq), epigenomic data (ChIP-seq, ATAC-seq, etc.)	Local web app, local server, local files	Navigation, annotations, zoom view, track configurations	Create from mouse selection	1D intervals created from mouse selection or files, with configurable displaying options, and scores	2D regions created from mouse selection or files, with configurable displaying options, and scores
Nucleome Browser [1]	✓	✓	✓	✓	Local server	Navigation, annotations		1D interval created from mouse selection	2D region created from mouse selection
WashU Epigenome Browser [2]	✓	✓		✓	Local web app, local files			1D interval created from jumping to a region using Genomic Region Locator	
HiGlass [3]		✓	✓	✓	Local web app, local server	Navigation	Create by editing view configuration, or create a new view and link back to the original view	Create by editing view configuration	Create by editing view configuration
3D Genome Browser [4]		✓	✓	✓					2D region created from mouse click
TADkit [5]	✓	✓		✓	Local web app				Cross line created from mouse click
HiC3D-Viewer [6]	✓	Only show as chromosome-wide, cannot zoom or pan	Only show between chromosome-wide, cannot zoom or pan		Local web app, local files			1D intervals created from BED file	2D regions created using mouse selection
HiCPlotter [7]		✓	Either whole genome or choose the end chromosome to plot	✓	Command line tool	Viewing region, annotations		1D intervals created from BED file	
pyGenomeTracks [8]		✓		✓	Command line tool	Viewing region		Vertical lines created from BED file	

[1] Zhu, X., et al. (2022). Nucleome browser: An integrative and Multimodal Data Navigation Platform for 4D nucleome. Nature Methods, 19(8), 911–913.

[2] Li, D., et al. (2022). WashU Epigenome Browser update 2022. Nucleic Acids Research, 50(W1), W774-W781.

[3] Kerpedjiev, P., et al. (2018). HiGlass: web-based visual exploration and analysis of genome interaction maps. Genome biology, 19(1), 1-12.

[4] Wang, Y., et al. (2018). The 3D Genome Browser: a web-based browser for visualizing 3D genome organization and long-range chromatin interactions. Genome biology, 19(1), 1-12.

[5] Serra, F., et al. (2017). Automatic analysis and 3D-modelling of Hi-C data using TADbit reveals structural features of the fly chromatin colors. PLoS computational biology, 13(7), e1005665.

[6] Djekidel, M. N., et al. (2017). HiC-3DViewer: a new tool to visualize Hi-C data in 3D space. Quantitative Biology, 5(2), 183-190.

[7] Akdemir, K. C., & Chin, L. (2015). HiCPlotter integrates genomic data with interaction matrices. Genome biology, 16(1), 1-8.

[8] Lopez-Delisle, L., et al. (2021). pyGenomeTracks: reproducible plots for multivariate genomic data sets. Bioinformatics, 37(3):422-423.
